# Tremella fuciformis beverage improves glycated hemoglobin A1c and waist circumference in overweight/obese prediabetic subjects: a randomized controlled trial

**DOI:** 10.1186/s40795-024-00842-0

**Published:** 2024-03-04

**Authors:** Sawika Gitsomboon, Ganista Ratanapornsompong, Boonsong Ongphiphadhanakul, Supranee Thongpradit, Suwannee Chanprasertyothin, La-or Chailurtkit, Hataikarn Nimitphong

**Affiliations:** 1 Division of Endocrinology and Metabolism, Department of Medicine, Faculty of Medicine Ramathibodi Hospital, Mahidol University, 270 Rama 6th Road, Ratchathewi, 10400 Bangkok, Thailand; 2 Research Center, Academic Affairs and Innovations, Faculty of Medicine Ramathibodi Hospital, Mahidol University, 270 Rama 6th Road, Ratchathewi, 10400 Bangkok, Thailand

**Keywords:** Tremella fuciformis, Prediabetes, Overweight, Obese, Insulin sensitivity, HbA1C

## Abstract

**Background:**

Prediabetes is increasing worldwide. Previous studies have demonstrated the potential of β-glucan derived from oat or barley to lower blood glucose, body weight, and plasma lipid levels. These findings offer a potentially attractive strategy for reducing the risk of diabetes in prediabetic individuals. However, the effects of β-glucan from Tremella fuciformis on glucose metabolism and anthropometric measurements in humans have yet to be studied. We hypothesized that β-glucan from Tremella fuciformis may improve metabolic parameters in subjects with prediabetes. This study aimed to investigate the effects of a once-daily beverage containing Tremella fuciformis (snow mushroom) on anthropometric measurements, metabolic biomarkers, and insulin sensitivity in overweight/obese subjects with prediabetes.

**Methods:**

In this double-blind RCT, 56 participants were randomly assigned to receive either a Tremella fuciformis beverage or placebo daily for 12 weeks. All parameters were assessed at baseline and after the intervention.

**Results:**

After 12 weeks, participants in the intervention group exhibited significant improvements in glycated hemoglobin A1c (HbA1C; 6.03 ± 0.26% at baseline vs. 5.96 ± 0.25% at 12 weeks, *p* = 0.047, Cohen’s d = 0.39) and waist circumference (95.2 ± 12.51 cm at baseline vs. 93.46 ± 11.48 cm at 12 weeks, *p* = 0.022, Cohen’s d = 0.45). There were no adverse events reported.

**Conclusion:**

This exploratory study demonstrated that Tremella fuciformis beverage consumption may improve HbA1C and waist circumference in overweight/obese prediabetic individuals. Further research, including larger-scale RCTs and mechanistic studies, is needed to confirm these findings and optimize the therapeutic potential of Tremella fuciformis derivatives in managing prediabetes and preventing type 2 diabetes.

**Trial registration:**

Registered in Thai Clinical Trials Registry (14/07/2021, TCTR20210714004).

## Highlights

Tremella fuciformis contains β-glucan and may have benefit in metabolic syndrome.

This is the first RCT exploring the effects of T. fuciformis in overweight/obese prediabetes subjects.

Twelve weeks of T. fuciformis consumption reduced HbA1C and waist circumference.

No adverse events were reported among subjects.

## Introduction

Prediabetes is defined as having blood glucose levels above normal but below the defined threshold of diabetes [1]. The prevalence of prediabetes was 10.6% in Thailand [2]. People with prediabetes are at an increased risk of developing type 2 diabetes (T2DM), nephropathy, neuropathy, retinopathy, and cardiovascular diseases [3–5]. Lifestyle interventions, such as exercise and weight loss, in combination with metformin, are often considered as strategies to delay or prevent the onset of diabetes in prediabetic individuals [6]. However, these interventions may be limited in real-world settings because of the requirement for high intensity and rigorous adherence. Recent evidence suggests that functional foods and their bioactive substances may be utilized as supplemental therapeutics for prediabetes and diabetes [7]. Consumption of functional foods offers a potentially attractive strategy for reducing the risk of diabetes in prediabetic individuals given that it can be more easily adhered to.

Tremella fuciformis, commonly known as snow mushroom, snow fungus, silver ear mushroom, or white jelly mushroom, is an edible mushroom with promising therapeutic properties [8]. Tremella polysaccharides are active substances found in the fruiting body, mycelium, and fermentation broth of the fungus [8]. Among these substances, 1,3-β-glucan constitutes up to 2.5% of the dry weight of the fungus [9]. Previous studies have demonstrated the potential of β-glucan derived from oats or barley to lower blood glucose, body weight, and plasma lipid levels in vitro, and in animal models [10–12]. Studies in humans have demonstrated that β-glucan obtained from oats or barley efficiently reduced blood glucose, insulin, and lipid levels in healthy subjects [13] and people with diabetes [14]. The β-glucan found in Tremella fuciformis differs from that in grains because of its β-1,6 linkage and branching structure, which may confer distinct medicinal properties [15]. However, in contrast to those derived from grains, the effects of β-glucan from Tremella fuciformis on glucose metabolism and anthropometric measurements in humans have not been investigated to date. There have only been a few studies examining the anti-obesity and lipid-lowering effects of this compound in mice and rats [16, 17]. Evidence suggested that Tremella fuciformis modulated gut microbiota as a possible mechanism [16, 17]..

We hypothesized that β-glucan from Tremella fuciformis may improve metabolic parameters in subjects with prediabetes. The objective of this study was to assess the impact of Tremella fuciformis beverage consumption on body measurements, metabolic biomarkers, and insulin sensitivity in overweight and obese subjects with prediabetes.

## Materials and methods

### Study participants

**Fig. 1:**
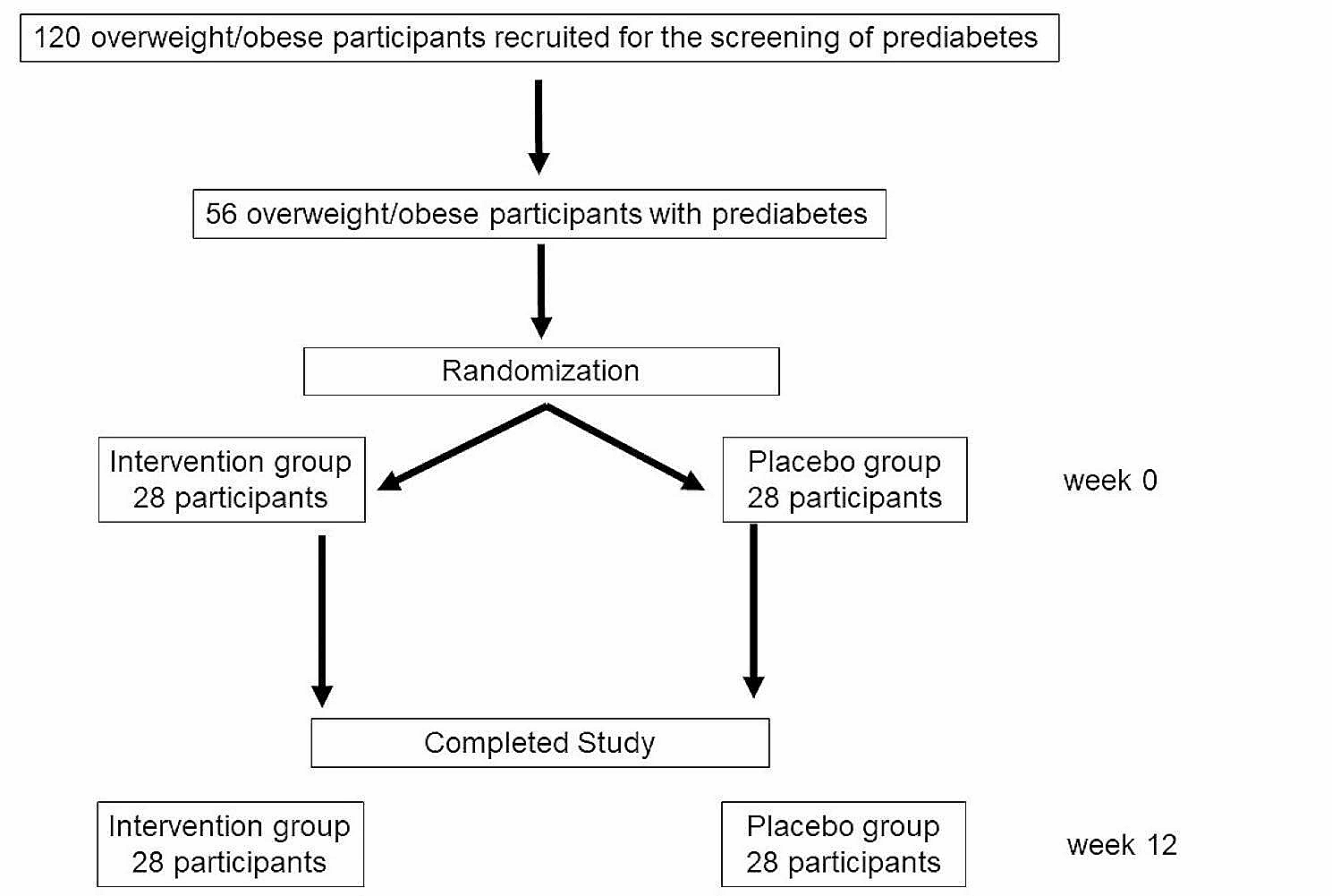
Flowchart. A total of 120 volunteers were screened for prediabetes and 56 eligible participants were enrolled. Participants were randomized into 2 groups; 28 in the intervention group and 28 in the placebo group and followed for 12 weeks. All participants were completed the study and assessed for clinical and biochemical parameters at week 0 and 12

One hundred twenty volunteers aged 15 to 60 years, from the outpatient clinic at the Internal Medicine Department at Ramathibodi Hospital, Thailand were recruited by advertisement for the screening of prediabetes between June 2021 and March 2022. Fifty-six eligible participants were enrolled (Fig. 1).

Inclusion criteria included prediabetic overweight/obese individuals aged 15–60 years who met the American Diabetes Association (ADA) criteria for prediabetes [glycated hemoglobin A1c (HbA1C) 5.7–6.4% and/or fasting plasma glucose (FPG) 100–125 mg/dL] [1] and had a body mass index (BMI) ≥ 23 kg/m^2^. Participants taking antihypertensive or lipid-lowering medications must not have had any medication adjustments within three months before recruitment. Exclusion criteria included refusal to participate, diabetes diagnosis or current use of antidiabetic agents, current use of medications affecting glucose metabolism (for example, steroid, tacrolimus, atypical antipsychotic drugs), pregnancy, or disability affecting activities of daily living.

The study protocol was approved by the institutional review board of the Faculty of Medicine, Ramathibodi Hospital, Mahidol University (MURA2020/939). The participants were informed about the details of this study, including the purpose, the procedure, risks, compensation for participation, participant rights, contact information, and a confidentiality agreement. After that, written informed consent was obtained from all participants. The clinical trial number is TCTR20210714004 (Thai Clinical Trials Registry).

### Study design

Participants were divided into two groups of 28 participants (Fig. 1). The first group received Tremella fuciformis beverage (containing 6.4 g of β-glucan in a 180 mL beverage) and the other received a similar-looking and tasting placebo drink of the same volume. The assignment of participants into treatments was done by computers (simple randomization), where the computer assigns each participant a code number and treatment group. The investigators and participants only knew the code number to avoid bias. Only the study coordinators were allowed to know the treatment received by each participant. Their role was to communicate this information to the company funding the research, specifically regarding the type of beverages administered. The CPF Food Research & Development Center Company Limited directly dispatched the beverages to the participants through mail every 4 weeks. Throughout the study, the list containing the names of participants and their corresponding code numbers remained confidential and undisclosed. This confidentiality measure was maintained until the completion of the study. Participants were requested to drink one bottle of beverage before dinner every day for 12 weeks (Fig. 1).

Participants had to visit the study clinic two times: before the start of the trial and at the end of the 12th week. At both visits, a medical history was taken and body weight, height, waist circumference, and blood pressure were measured. Participants were asked not to modify their lifestyle habits nor their medication unless directed to do so by physician throughout the study period. Participants had to consume a regular diet containing at least 150 g of carbohydrate per day for three days prior to the test and fast for at least ten hours before each visit. 75 oral glucose tolerance test (OGTT) was assessed at baseline and 12th week. Blood samples were taken to measure plasma glucose and insulin at 0, 30, 60, 90 and 120 min. Insulin resistance and sensitivity were assessed using the Matsuda index [18], homeostatic model assessment-insulin resistance (HOMA-IR), the insulinogenic index, and the insulin disposition index. HbA1C and lipid profiles were also measured at both visits. The physical activity and sedentary behavior were assessed by the Global Physical Activity Questionnaire (GPAQ) version 2 [19]. Compliance was confirmed using the participants’ daily self-monitoring logs and monthly telephone follow-ups.

### The beverages’ preparation

Tremella fuciformis was obtained from a local supermarket in Thailand. The samples were soaked and boiled with boiling water with a mushroom:water ratio of 15:85, (w/v) at 95°C for 10 min. The aqueous solution containing the extract was mixed with 10% monk fruit solution in water and sterilized by retort sterilization. To prepare a placebo drink, a mixture of 10% monk fruit solution and water was combined to equal the volume of Tremella fuciformis drink (180 mL) and sterilized by retort sterilization.

### Nutritional composition of beverages

One bottle (180 mL) of Tremella fuciformis beverage contains 0% protein, 0% fat, 0% carbohydrate, 3% dietary fiber, and 1% sodium of the Thai Recommended Daily Intakes for adults and provides 0 kcal of energy. No vitamins (A, B1 and B2) and minerals (calcium and iron) are found in the content. The placebo beverage contains the same volume and components as the Tremella fuciformis beverage, except for Tremella fuciformis.

### The determination of β-glucan and other bioactive compounds in Tremella fuciformis

The β -glucan content of Tremella fuciformis and their fractions were determined by the Thailand Institute of Scientific and Technological Research using a yeast and mushroom beta-glucan assay kit (Megazyme, code K-YBGL). In this study, the Tremella fuciformis beverage (180 mL) contained 15% Tremella fuciformis (6.4 g of β-glucan), 0.08% Luo Han Guo, and 0.04% licorice.

Total phenolic contents and antioxidant activities of Tremella fuciformis were investigated using the Folin-Ciocalteu method and DPPH method, respectively. In this study, the polyphenols content was 132.88 ± 8.95 µg of gallic acid equivalents /ml of sample and the antioxidant capacity was 23.37 ± 12.24 µg Trolox equivalents (TE)/ml of sample.

### Outcomes

Primary outcomes were changes in anthropometric, metabolic, and biochemical parameters at the end of the study. Secondary outcomes were adverse effects caused by the treatment and compliance.

### Statistical analysis

The intention-to-treat analysis was used to assess treatment efficacy. Continuous variables with normal distribution were reported as mean ± standard deviation (SD), while those with non-normal distribution were reported as median and interquartile range. The Kolmogorov-Smirnov normality test was used to determine whether variables were normally distributed. To compare the difference in clinical characteristics at baseline between the intervention and the placebo groups, we used the Independent-Samples T test, the Mann-Whitney test, and the Chi-square test, as appropriate. Changes in demographic, anthropometric, metabolic, and biochemical parameters after intervention in each group were assessed using paired T-tests. Results were considered significant if the p-value was < 0.05. The Wilcoxon test was used for parameters that were not normally distributed. Cohen’s d (based on differences) was calculated to report the effect size of the differences. Because no previous study has addressed the same issue, the target sample size was to recruit at least 10–12 participants per arm, as previously suggested [20]. Statistical analyses were performed using SPSS software version 28.0 (IBM, USA).

## Results

### Baseline characteristics

A total of 56 participants were recruited and randomized into the intervention and placebo groups (Fig. 1). The mean age of participants was 40.63 ± 8.69 years, with 46.4% (*n* = 26) being male. The mean body weight and BMI were 84.72 ± 18.24 kg and 31.68 ± 6.71 kg/m^2^, respectively. The mean waist circumference was 102.44 ± 13.32 cm for males and 96.55 ± 12.48 cm for females, with an average waist/hip ratio of 0.87 ± 0.07. The mean baseline FPG value was 93.59 ± 10.68 mg/dL, and baseline HbA1C was 6 ± 0.15%. Table 1 shows the baseline characteristics of participants in both groups. All parameters were similar between the two groups.

**Table 1:** Baseline characteristics of overweight/obese prediabetic participants in the intervention (Tremella fuciformis beverage) and placebo group

Characteristics	Intervention group (*n* = 28)	Placebo group (*n* = 28)	P-value
**Demographics and anthropometric parameters**			
Age (years)	40.64 ± 8.39	40.61 ± 9.14	0.988^a^
Male gender, number (%)	12(42.9)	14(50)	0.789^b^
Family history of diabetes, number (%)	11(39.3)	8(28.6)	0.573^b^
Body weight (kg)	81.56 ± 16.14	87.88 ± 19.92	0.198^a^
BMI (kg/m^2^)	30.89 ± 5.76	32.48 ± 7.57	0.380^a^
Waist circumference (cm)			
Male	102.46 ± 14.65	102.43 ± 12.64	0.996^a^
Female	89.75 ± 7.1	93.36 ± 11.06	0.291^a^
Waist/hip ratio	0.87 ± 0.09	0.87 ± 0.06	0.828^a^
Current smoking, number (%)	2(7.1)	3(10.7)	1.000^b^
Current alcohol use, number (%)	3(10.7)	7(25)	0.295^b^
Underlying disease, number (%)			
Dyslipidemia	4(14.3)	5(17.9)	1.000^b^
Hypertension	4(14.3)	8(28.6)	0.329^b^
Others	5(17.9)	7(25)	0.746^b^
**Metabolic and other biochemical parameters**			
FPG (mg/dL)	94.68 ± 11.21	92.5 ± 10.21	0.451^a^
HbA1C (%)	6.03 ± 0.16	5.96 ± 0.13	0.064^a^
2 h-PG (mg/dL)	143.5 ± 30.21	148.64 ± 26.12	0.499^a^
Total cholesterol (mg/dL)	201.71 ± 31.3	206.82 ± 54.75	0.910^a^
HDL cholesterol (mg/dL)	50.93 ± 7.12	48.11 ± 10.59	0.247^a^
LDL cholesterol (mg/dL)	129.68 ± 31.6	139.71 ± 39.53	0.299^a^
Triglyceride (mg/dL)	142.5 ± 86.6	134.61 ± 89.39	0.738^a^
**Physical activity**			
Moderate-to-vigorous activity (METs)	0 (0-1140)	0 (0-1650)	0.898^c^
Sedentary time (hours/day)	2.36 ± 3.9	2.09 ± 2.38	0.757^a^

Data were presented as mean ± SD

BMI: body mass index, FPG: fasting plasma glucose, 2 h-PG: 2-h plasma glucose, HbA1C: glycated hemoglobin A1c, HDL: high-density lipoprotein, LDL: low-density lipoprotein, METs: Metabolic equivalent of tasks

^a^independent-Samples T Test

^b^the Chi-square test

^c^Mann-Whitney U

### Changes in anthropometric parameters

After 12 weeks of intervention, anthropometric, metabolic, and biochemical parameters were assessed and are presented in Table 2. In the intervention group, there was a significant reduction in waist circumference (95.2 ± 12.51 cm at baseline vs. 93.46 ± 11.48 cm at 12 weeks, *p* = 0.022, Cohen’s d = 0.45). However, there were no changes in body weight, BMI and waist/hip ratio. In contrast, there were no changes in all anthropometric parameters in the placebo group.

**Table 2:** Compared parameters at baseline and 12 weeks of overweight/obese prediabetic participants in the intervention (Tremella fuciformis beverage) and placebo group

Characteristics	Intervention group (*n* = 28)		P-value	Placebo group (*n* = 28)		P-value
	At week 0	At week 12		At week 0	At week 12	
**Demographic and anthropometric parameters**						
Body weight (kg)	81.56 ± 16.14	80.80 ± 16.15	0.140^a^	87.88 ± 19.92	88.63 ± 21.77	0.334^a^
BMI (kg/m^2^)	30.89 ± 5.76	30.66 ± 5.85	0.165^a^	32.48 ± 7.57	31.89 ± 6.11	0.622^a^
Waist circumference (cm)	95.20 ± 12.51	93.46 ± 11.48	0.022^a^	97.89 ± 12.54	98.54 ± 14.36	0.395^a^
Waist/hip ratio	0.87 ± 0.09	0.86 ± 0.08	0.265^a^	0.87 ± 0.06	0.87 ± 0.06	0.297^a^
**Metabolic and other biochemical parameters**						
FPG (mg/dL)	94.68 ± 11.21	94.39 ± 10.14	0.888^a^	92.5 ± 10.21	94.21 ± 9.27	0.273^a^
HbA1C (%)	6.03 ± 0.16	5.96 ± 0.25	0.047^a^	5.96 ± 0.13	5.95 ± 0.33	0.891^a^
2 h-PG (mg/dL)	143.5 ± 30.21	135.32 ± 34.4	0.164^a^	148.64 ± 26.12	144.68 ± 32.64	0.451^a^
Matsuda index	2.93 (0.60-22.12)	3.63 (0.63–37.77)	0.133^b^	3.83 (1.10-19.14)	2.59 (0.40-28.11)	0.198^b^
HOMA-IR	3.69 (0.08–13.88)	3.22 (0.30-25.56)	0.900^b^	3.16 (0.83–11.32)	3.52 (0.07–47.19)	0.236^b^
Insulinogenic index	0.43 (-0.88-2.45)	0.25 (-0.31-4.73)	0.864^b^	0.47 (-0.40-3.24)	0.45 (-0.39-2.41)	0.710^b^
Disposition index	0.81 (-1.77-3.06)	0.46 (-0.627-6.26)	0.973^b^	0.59 (-1.05-6.92)	1.2 (-1.31-5.74)	0.741^b^
Total cholesterol (mg/dL)	201.71 ± 31.3	192.86 ± 33.86	0.200^a^	200.54 ± 45.31	202.21 ± 55.3	0.869^a^
HDL cholesterol (mg/dL)	50.93 ± 7.12	51.61 ± 10.42	0.527^a^	48.11 ± 10.59	47.68 ± 10.38	0.723^a^
LDL cholesterol (mg/dL)	129.68 ± 31.6	126.11 ± 35.38	0.508^a^	139.71 ± 39.53	136.07 ± 46.79	0.724^a^
Triglycerides (mg/dL)	121.0 (54–445)	115.5 (54–425)	0.330^b^	107.5 (43–441)	103.5 (48–619)	0.399^b^

Data were presented as mean ± SD or IQR

BMI: body mass index, FPG: fasting plasma glucose, 2 h-PG: 2-h plasma glucose, HbA1C: glycated hemoglobin A1c, HOMA-IR: homeostatic model assessment-insulin resistance, HDL: high-density lipoprotein, LDL: low-density lipoprotein

^a^paired Student’s t-test

^b^Wilcoxon Signed Ranks Test

### Changes in metabolic and biochemical parameters

At 12 weeks, there was a significant decrease in HbA1C in the intervention group (6.03 ± 0.16 at baseline vs. 5.96 ± 0.25 at 12 weeks, *p* = 0.047, Cohen’s d = 0.39). FPG, 2-hour plasma glucose, Matsuda index, HOMA-IR, insulinogenic index, disposition index, and lipid profile did not change in the intervention group. In the placebo group, no changes were observed in any metabolic or biochemical parameters. The results are shown in Table 2.

### Side effects and compliance

There were no adverse events reported by participants in either group. The compliance of 51 of 56 participants could be examined, and the average compliance assessed by the self-monitoring logs was 98.81 ± 3.16%.

## Discussion

This is an exploratory study assessing the effect of Tremella fuciformis on improving some metabolic biomarkers among overweight/obese prediabetic subjects. Participants who consumed one bottle of Tremella fuciformis beverage daily for 12 weeks exhibited statistically significant improvements in HbA1C and waist circumference. The effect size for the differences in HbA1c and waist circumference were considered a small to medium effect. However, other metabolic and biochemical parameters remained unchanged after the 12-week intervention. This investigation was the first to explore the effects of Tremella fuciformis consumption on anthropometric and metabolic parameters in subjects with prediabetes. No adverse effects were reported by participants who consumed the Tremella fuciformis beverage. A key strength of our trial was its double-blind randomized controlled design, which is the first to evaluate changes in insulin sensitivity and resistance in prediabetic subjects. Further research with varying amounts of β-glucan and longer study durations may help clarify the beneficial effects of β-glucans derived from Tremella fuciformis on glucose and lipid metabolism.

In recent years, there have been several studies investigating the effects of functional foods and bioactive compounds on prediabetic and diabetic individuals [21, 22]. While our study is the first to specifically examine the impact of Tremella fuciformis beverage consumption, previous research has focused on β-glucans derived from other sources, such as oats and barley [23–25]. These studies have consistently demonstrated beneficial effects of β-glucans on plasma glucose and lipid levels in humans. For instance, a meta-analysis conducted by Whitehead et al. [26] revealed that oat-derived β-glucan consumption resulted in significant reductions in total and LDL cholesterol levels. Similarly, a meta-analysis by AbuMweis et al. [27] found that barley-derived β-glucan possesses lipid-lowering capacity. Additionally, a randomized controlled trial by Tappy et al. [25] reported that consuming a diet rich in whole grains, including β-glucans, significantly reduced HbA1C levels in individuals with type 2 diabetes mellitus. While these studies provide valuable insights into the potential benefits of β-glucans derived from grains, it is important to note that the Tremella fuciformis-derived β-glucans differ in structure, which may contribute to distinct physiological effects. Alterations in gut microbiota may partly be responsible for the positive effects of Tremella fuciformis-derived β -glucans on metabolic indicators [16, 17, 28]. Moreover, the phenolic components of the mushroom which possess metabolic regulatory effects as well as antioxidant activity may also contribute to this benefit [29].

Despite the promising findings, our study has several limitations that should be acknowledged. First, the small sample size may have limited our ability to detect significant differences in other metabolic and biochemical parameters, reducing the statistical power of the study. Furthermore, a small sample size may result in imbalances in participant baseline characteristics (selection bias). Second, participant compliance was assessed using phone tracing and self-declaration, which could be subject to reporting bias and may not accurately reflect true compliance levels. Additionally, the 12-week intervention period may not have been long enough to detect more substantial changes in metabolic and biochemical parameters, particularly given the chronic nature of prediabetes and type 2 diabetes mellitus. Furthermore, our study did not include a dose-response analysis, which could provide insight into the optimal amount of β-glucan required to elicit significant improvements in glucose metabolism and anthropometric measurements. We did not measure β-glucan levels in this study. Lastly, the lack of mechanistic investigations prevents a clear understanding of the underlying biological pathways through which Tremella fuciformis-derived β-glucans may exert their beneficial effects. Future studies should address these limitations by employing larger sample sizes, more rigorous compliance assessment methods, longer intervention durations, dose-response analyses, and in-depth mechanistic evaluations.

## Conclusion

This exploratory study provides preliminary evidence that Tremella fuciformis beverage consumption may improve HbA1C and waist circumference in overweight and obese prediabetic individuals. Further research is needed to confirm these findings and optimize the therapeutic potential of Tremella fuciformis derivatives in treating prediabetes and preventing type 2 diabetes mellitus.

## Data Availability

The raw data supporting the conclusions of this article will be made available by the authors upon request to hataikarnn@hotmail.com, without undue reservation.

## Abbreviations

2h-PG: 2-h plasma glucose
ADA: American Diabetes Association
BMI: Body mass index
FPG: Fasting plasma glucose
HbA1C: glycated hemoglobin A1c
HDL: high-density lipoprotein
HOMA-IR: homeostatic model assessment-insulin resistance
LDL: low-density lipoprotein
METs: Metabolic equivalent of tasks
OGTT: oral glucose tolerance test
PreDM: prediabetes
RCT: randomized controlled trial
